# CircRNA circuits orchestrate lactate metabolism in gastrointestinal cancer progression

**DOI:** 10.1515/biol-2025-1351

**Published:** 2026-07-29

**Authors:** Qingjuan Chen, Haiqing Zhou, Zhen Shen, Yadong Zhou, Jianlong Ding, Changhu Duan, Xia Cao, Ying Liu, Ling Wang, Wenjing He

**Affiliations:** Department of Oncology, 3201 Hospital, Hanzhong, 723000, China; Department of Infection Prevention and Control, 3201 Hospital, Hanzhong, 723000, China; Department of Gastrointestinal Surgery, 3201 Hospital, Hanzhong, 723000, China; Department of Hepatobiliary Surgery, 3201 Hospital, Hanzhong, 723000, China; School of Biological Science and Engineering, Shaanxi University of Technology, Hanzhong, 723000, China; Department of Oncology, Xianyang Center Hospital, Xi’an, 712000, Shaanxi, China

**Keywords:** circular RNAs (circRNAs), lactate metabolism, gastrointestinal cancers, Warburg effect, tumor microenvironment

## Abstract

Circular RNAs (circRNAs) have emerged as pivotal modulators of cancer metabolic reprogramming, orchestrating glycolysis and lactate metabolism to fuel malignant progression. Acting predominantly as competing endogenous RNAs (ceRNAs), circRNAs sequester tumor-suppressor microRNAs, thereby upregulating glycolytic enzymes, transporters, and key metabolic regulators. Across gastric, colorectal, esophageal, pancreatic, and hepatocellular carcinomas, oncogenic circRNAs converge to enhance glucose uptake, lactate production, and ATP generation, sustaining proliferation, metastasis, stemness, and therapy resistance. In contrast, tumor-suppressive circRNAs attenuate glycolytic flux, depriving cancer cells of energy and biosynthetic precursors. Mechanistic diversity-spanning circRNA-microRNA-hexokinase 2 and circRNA-microRNA-lactate dehydrogenase A axes, as well as hypoxia-induced circRNA signaling networks-underscores their cancer-type specificity and potential as precision oncology targets. Despite promising diagnostic and therapeutic implications, challenges remain in delivery, specificity, and functional annotation. Future advances will rely on multi-omics integration, high-throughput functional screening, and validation in patient-derived models to identify clinically actionable circRNA-metabolism regulatory circuits. Mapping these interactions offers not only biomarkers for prognosis and therapy response but also potential intervention points to rewire tumor metabolism.

## 1 Introduction

Metabolic reprogramming is a hallmark of cancer, enabling malignant cells to sustain rapid proliferation and adapt to hostile microenvironments [1]. One of the well-characterized metabolic alterations in tumors is the Warburg effect, wherein cancer cells preferentially utilize glycolysis for ATP production, even in the presence of oxygen, leading to excessive lactate accumulation [2]. Historically, lactate was widely considered a mere metabolic waste product with minimal biological function. Early research viewed it as a by-product of anaerobic metabolism, contributing only to cellular acidosis and muscle fatigue [3].

However, recent studies have revealed that lactate plays a far more dynamic role in cellular physiology and tumor biology. In the tumor microenvironment (TME), lactate serves as a critical metabolite that not only fuels surrounding cancer cells through metabolic symbiosis but also functions as a signaling molecule that influences gene expression, epigenetic modifications, and intercellular communication [4], [5], [6]. Lactate can be taken up by oxidative tumor cells and stromal cells via monocarboxylate transporters (MCTs), thereby providing an alternative energy source and supporting mitochondrial respiration [7]. This metabolic flexibility enables tumor cells to thrive under nutrient-limited or hypoxic conditions.

Lactate dehydrogenase (LDH) catalyzes the reversible conversion between pyruvate and lactate, linking glycolysis to cellular metabolic status. Increased LDH activity is associated with enhanced tumor cell metabolic activity and proliferation, and LDH-based assays have been used to evaluate proliferation-related changes in tumor cell culture systems [8], 9]. Extracellular LDH release reflects membrane damage, cytotoxic stress, or cytokine-induced cell injury, supporting its use as an indicator of cell viability and membrane integrity [10]. Inflammatory cytokines such as TNF-α can also alter LDH isoenzyme activity profiles in lymphoma-associated lymphocytes, suggesting that inflammatory signaling may modulate LDH-related metabolic states in tumor-associated cells [11]. Clinically, LDH is recognized as an accessible biomarker for tumor diagnosis, prognosis, and disease monitoring in multiple cancers, including digestive system malignancies [12].

Moreover, lactate accumulation exerts profound effects on the TME by acidifying the extracellular space, which promotes angiogenesis, immune suppression, and metastasis [13], 14]. It also stabilizes hypoxia-inducible factor-1α (HIF-1α), further driving glycolytic gene expression and reinforcing the Warburg phenotype [15]. Additionally, lactate acts as a substrate for histone lactylation, a recently discovered post-translational modification that modulates gene transcription and contributes to tumorigenesis [16]. These findings have shifted the perception of lactate from a passive metabolic by-product to a central orchestrator of cancer development, progression, and therapeutic resistance.

Circular RNAs (circRNAs), a class of endogenous non-coding RNAs, have garnered increasing attention due to their unique covalently closed-loop structure, which confers high stability and regulatory potential [17]. CircRNAs function through diverse mechanisms, including acting as microRNA (miRNA) sponges, interacting with RNA-binding proteins, and modulating gene transcription. Emerging evidence indicates that circRNAs contribute to the progression of gastrointestinal (GI) cancers by regulating epithelial-mesenchymal transition (EMT), oncogenic signaling pathways, and tumor-microenvironment interactions. Multiple circRNAs – including circPVT1, circGOT1, circVPS33B, circMET, circPGPEP1, and circ-E-Cad - have been reported to modulate EMT transcription factors and metastatic phenotypes across GI cancers [18]. These findings suggest that circRNAs function as critical regulatory nodes in GI tumor biology beyond their roles in conventional gene regulation.

Recent studies further indicate that circRNAs play a pivotal role in metabolic reprogramming, particularly in regulating glycolysis and lactate metabolism [19]. By targeting key metabolic enzymes and transporters, circRNAs contribute to the metabolic plasticity of cancer cells, thereby facilitating tumor progression and therapeutic resistance [20]. These findings underscore the significance of circRNAs as critical regulators of cancer metabolism and highlight their potential as novel diagnostic biomarkers and therapeutic targets in oncology.

In GI cancers, including liver, gastric, colorectal, esophageal, and pancreatic cancers, dysregulated lactate metabolism is closely linked to tumor aggressiveness and poor prognosis [21]. The involvement of circRNAs in this process highlights a critical regulatory axis with potential clinical implications. In this review, we comprehensively summarize the interplay between circRNAs and lactate metabolism in GI cancers, emphasizing their mechanistic roles, oncogenic impact, and potential as therapeutic targets.

## 2 The role of lactate metabolism in GI cancers

Lactate metabolism represents a hallmark of metabolic reprogramming in GI cancers. A defining feature of this reprogramming is the Warburg effect, whereby cancer cells preferentially engage glycolysis over oxidative phosphorylation for ATP production, even in the presence of sufficient oxygen. This metabolic bias leads to excessive lactate accumulation and substantial remodeling of the TME, thereby promoting tumor progression through immunosuppression, metastasis, and therapy resistance [2].

### 2.1 Glycolysis and the Warburg effect in GI cancers

The Warburg effect, first observed by Otto Warburg, describes the ability of cancer cells to increase glucose uptake and convert pyruvate into lactate despite adequate oxygen availability. Although this aerobic glycolysis is less efficient for ATP generation, it confers growth advantages by supplying biosynthetic intermediates required for nucleotide, amino acid, and lipid synthesis. In addition, the process regenerates NAD^+^, thereby maintaining redox balance critical for sustaining rapid cell proliferation [22], 23].

**Figure 1:**
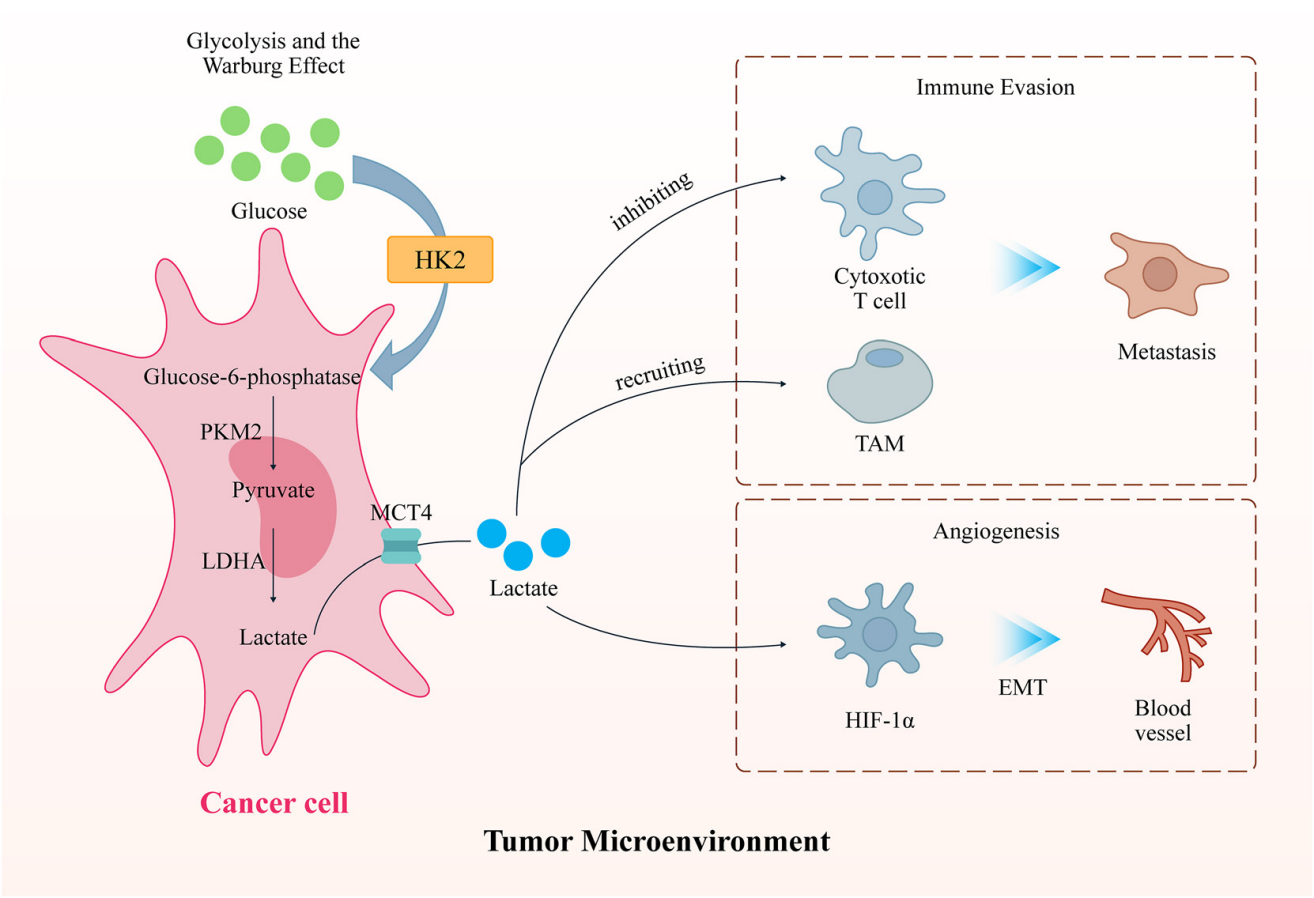
Warburg effect-driven lactate metabolism in cancer cells and its impact on the tumor microenvironment (TME). In cancer cells, glucose is taken up and metabolized through glycolysis. Hexokinase 2 (HK2) initiates glycolysis by phosphorylating glucose to glucose – 6 – phosphate. Pyruvate kinase M2 (PKM2) then converts intermediates to pyruvate at a low catalytic efficiency, which is further reduced to lactate by lactate dehydrogenase A (LDHA). Lactate is transported out of cancer cells via monocarboxylate transporter 4 (MCT4). Once in the TME, lactate induces acidification, which drives multiple pro-tumor processes. It promotes immune evasion by inhibiting cytotoxic T cells and recruiting tumor-associated macrophages (TAMs), facilitates metastasis, and contributes to angiogenesis through hypoxia – inducible factor – 1α (HIF – 1α) – mediated epithelial-mesenchymal transition (EMT), ultimately creating a favorable microenvironment for tumor progression.

Several glycolytic enzymes are aberrantly upregulated in GI cancers to maintain this metabolic phenotype (Figure 1). Hexokinase 2 (HK2) catalyzes the first committed step of glycolysis, phosphorylating glucose to glucose-6-phosphate using ATP, thereby driving glucose flux into glycolysis and anabolic pathways such as nucleotide biosynthesis [23]. HK2 also binds to the mitochondrial outer membrane via voltage-dependent anion channel (VADC) protein, stabilizing mitochondrial integrity and inhibiting apoptosis. In GI cancers, HK2 overexpression is common: in colorectal cancer (CRC), it correlates with increased proliferation and poor prognosis, and knockout enhances 5-fluorouracil sensitivity [24], 25]; in hepatocellular carcinoma (HCC), depletion of HK2 reduces glycolysis and synergizes with sorafenib [26]; in gastric cancer (GC), HIF-1α directly upregulates HK2, promoting tumor growth [27]. This highlights HK2 as a metabolic gatekeeper whose dual role in energy metabolism and apoptosis suppression makes it a compelling therapeutic target in GI cancers.

Pyruvate kinase M2 (PKM2), a key rate-limiting enzyme, catalyzes the final step of glycolysis by converting phosphoenolpyruvate to pyruvate with concomitant ATP production. Its low catalytic efficiency causes glycolytic intermediates to accumulate, thereby fueling anabolic pathways [22]. In hepatocellular carcinoma, PKM2 modulates mitochondrial dynamics and glycolysis, with long non-coding RNAs (lncRNAs) such as LINC01554 upregulating PKM2 through autophagy-miRNA pathways [28]. In CRC, lncRNA FEZF1-AS1 stabilizes PKM2, enhancing both its kinase and pyruvate kinase activities to promote tumor progression [29]. In pancreatic cancer (PC), PKM2 overexpression is associated with M2 macrophage infiltration and poor prognosis [30]. Pharmacological PKM2 inhibitors (e.g., Benserazide, C3k) have been shown to suppress glycolysis and tumor growth in preclinical models. These findings position PKM2 not only as a metabolic enzyme but also as a signaling hub that links metabolic rewiring to oncogenic transcriptional programs.

Lactate dehydrogenase A (LDHA) catalyzes the reversible conversion of pyruvate to lactate, coupled with NADH oxidation to NAD^+^, thereby sustaining glycolytic flux and maintaining NADH/NAD^+^ homeostasis in tumor cells [7], 31]. LDHA is frequently overexpressed in digestive system malignancies: in pancreatic cancer, it is upregulated throughout carcinogenesis to support hypoxic adaptation [32], 33]. In gastric cancer, it is elevated in HER2-positive tumors, driving aerobic glycolysis [34]; in hepatocellular carcinoma, LDHA depletion triggers mitochondrial dysfunction, reactive oxygen species (ROS) accumulation, Ca^2+^ overload, and apoptosis via cytochrome C release [35]. High LDHA levels are associated with poor prognosis, tumor progression, and chemoresistance. Thus, LDHA serves as a metabolic accelerator that fuels both energy production and the creation of a pro-tumorigenic microenvironment, underscoring its value as a prognostic marker and drug target.

A coordinated transcriptional program, largely driven by HIF-1α and c-Myc, underpins the upregulation of HK2, PKM2, and LDHA, ensuring sustained lactate production and export. HIF-1α, stabilized under the hypoxic conditions prevalent in solid tumors, directly transactivates the promoters of HK2, PKM2, and LDHA, thereby enhancing glucose phosphorylation, pyruvate-to-lactate conversion, and redox homeostasis [36]. In hepatocellular carcinoma, HIF-1α not only elevates HK2 expression but also facilitates its binding to VDAC1, supporting ATP synthesis and anti-apoptotic signaling. HIF-1α-driven PKM2 upregulation promotes glycolytic flux and nuclear PKM2 translocation, where it acts as a co-activator of oncogenic transcription factors. By increasing LDHA expression, HIF-1α accelerates lactate production, creating a positive feedback loop that reinforces the Warburg effect, tumor proliferation, angiogenesis, and drug resistance [26], 37], 38].

c-Myc, a master regulator of cellular metabolism, similarly drives aerobic glycolysis by transcriptionally activating HK2, PKM2, and LDHA [39], [40], [41]. In hepatocellular carcinoma, c-Myc upregulates HK2 to enhance glucose phosphorylation, binds to the PKM2 promoter to increase the PKM2/PKM1 ratio, and promotes PKM2 nuclear translocation for β-catenin co-activation. Moreover, c-Myc elevates LDHA expression, accelerating lactate production and reinforcing glycolytic dependency. These activities form self-reinforcing loops that sustain glycolytic gene expression, anabolic growth, and aggressive tumor behavior.

The resulting lactate-rich, acidic TME not only fuels tumor metabolism but also promotes angiogenesis, immune evasion, and stromal remodeling, ultimately establishing a microenvironment that supports tumor survival, progression, and resistance to therapy [42]. This intricate metabolic and transcriptional interplay underscores the potential of targeting glycolysis-related enzymes and their upstream regulators as a therapeutic avenue in GI cancers.

### 2.2 Lactate-induced histone lactylation in tumor progression

In GI cancers, lactate accumulation within the TME is primarily driven by the enhanced activity of LDHA, which catalyzes the conversion of pyruvate to lactate, and MCT1 and MCT4, which mediate lactate efflux and uptake. These transporters are transcriptionally activated by the oncogenic transcription factors HIF-1α and c-Myc, both frequently overexpressed in GI tumors [43]. Elevated lactate levels acidify the extracellular space, promoting tumor aggressiveness, angiogenesis, and resistance to therapy [44]. This acidic TME also alters cancer cell signaling and reduces drug uptake, posing a major barrier to effective treatment.

Beyond its metabolic function, lactate serves as a substrate for lactylation, a post-translational modification in which lactate-derived acyl groups are added to lysine residues on histones and other proteins. First identified in macrophages, histone lactylation has since been observed in multiple tumor models [16]. This epigenetic mechanism regulates gene expression, activates oncogenes, and supports pro-tumor inflammatory or immunosuppressive programs. In CRC, histone lactylation enhances Wnt/β-catenin signaling, thereby sustaining tumor stemness and chemoresistance [45]. Specific examples include KAT8-mediated lactylation of eukaryotic elongation factor 1 alpha 2(eEF1A2) at K408, which boosts GTPase activity to accelerate protein synthesis, and histone deacetylase 1 (HDAC1) lactylation at K412, which confers ferroptosis resistance. Additionally, lactate-induced acidosis facilitates immune evasion, further driving CRC progression [46].

In HCC, the splicing factor SRSF10 stabilizes MYB mRNA, upregulating glycolytic enzymes ((Glucose transporter 1, GLUT1), HK1, LDHA) and increasing lactate production. The accumulated lactate induces histone H3K18 lactylation, which in turn further elevates SRSF10 expression, forming a positive feedback loop. Lactate also promotes macrophage H3K18 lactylation, driving M2 polarization and suppressing CD8^+^ T cell activity, thus fostering an immunosuppressive TME, accelerating tumor progression, and contributing to anti-PD-1 resistance [47].

In GC, hyperactive glycolysis leads to high lactate levels, which correlate with increased histone H3K18 lactylation and poor overall and recurrence-free survival. This modification accelerates glycolysis, establishing a vicious cycle that fuels tumor growth. Lactate also enhances DNA repair by promoting Nijmegen breakage syndrome protein 1(NBS1) lactylation at K388, facilitating MRN complex formation and DNA double-strand break repair, thereby contributing to chemoresistance. Furthermore, alanyl-tRNA synthetase 1 (AARS1) acts as a lactyltransferase that senses intracellular lactate, translocates to the nucleus, and lactylates YAP-TEAD1, activating the Hippo pathway to drive proliferation [46].

Collectively, lactylation links metabolic reprogramming to epigenetic regulation in GI cancers, influencing stemness, drug resistance, and immune evasion. This dual metabolic-epigenetic axis represents both a biomarker of tumor aggressiveness and a promising target for therapeutic intervention through small-molecule inhibitors or metabolic modulation strategies.

### 2.3 Influence of lactate on the tumor microenvironment, immune evasion, and metastasis

**Figure 2:**
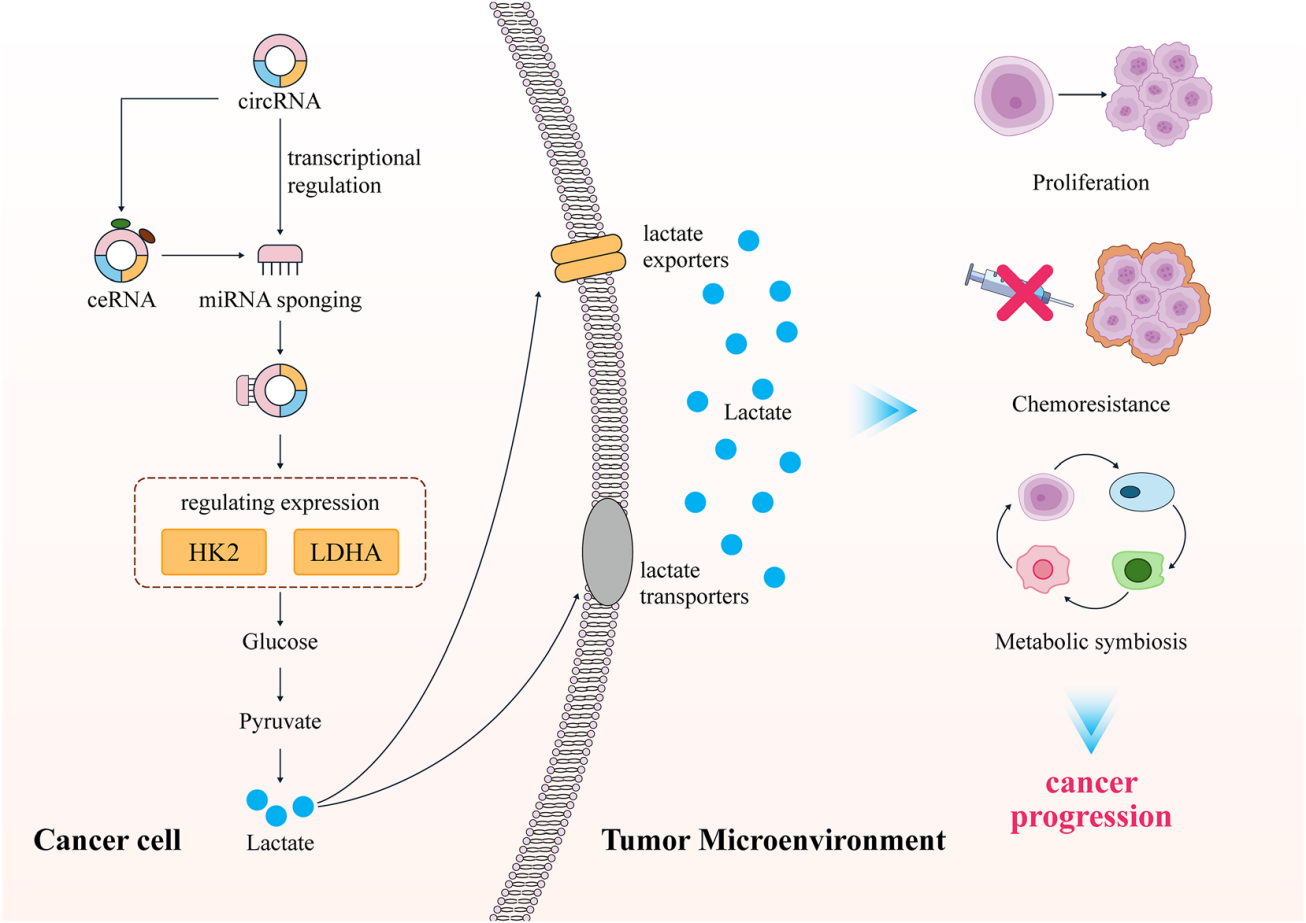
The regulatory role of circRNAs in lactate metabolism and their impact on the tumor microenvironment. CircRNAs modulate glycolysis and lactate production through transcriptional regulation and by acting as competing endogenous RNAs (ceRNAs) that sponge miRNAs, thereby relieving repression of key glycolytic enzymes such as HK2 and LDHA. Enhanced glycolytic flux increases glucose uptake, pyruvate generation, and conversion to lactate, which is exported via lactate transporters and exporters. Elevated extracellular lactate contributes to tumor cell proliferation, chemoresistance, and metabolic symbiosis within the tumor microenvironment, promoting cancer progression.

Tumor progression is shaped by complex interactions among cancer cells and diverse cellular components of the TME, including tumor-infiltrating lymphocytes, macrophages, myeloid-derived suppressor cells, fibroblasts, endothelial cells, and stromal cells (Figure 2). These cells produce cytokines, chemokines, growth factors, and metabolites that reshape immune activity, reinforce immunosuppression, and generate broad metabolic effects within the tumor microenvironment [48]. The high levels of lactate in the TME not only reflect the metabolic activity of tumor cells but also actively contribute to shaping a permissive environment for cancer progression. Lactate exerts immunosuppressive effects by directly inhibiting the function of cytotoxic T lymphocytes (CTLs) and natural killer (NK) cells, which are critical for anti-tumor immunity [49]. Elevated extracellular lactate suppresses T-cell proliferation and cytokine production, particularly interferon-gamma (IFN-γ), thereby reducing immune-mediated tumor clearance [50]. Simultaneously, lactate promotes the expansion and activation of regulatory T cells (Tregs) and myeloid-derived suppressor cells (MDSCs), which further dampen the immune response within the TME [51].

Lactate also supports angiogenesis through the stabilization of HIF-1α and upregulation of vascular endothelial growth factor (VEGF). Lactate, taken up by endothelial cells via MCT1, activates HIF-1 in normoxic conditions. This increases VEGF receptor 2 and basic fibroblast growth factor (bFGF) expression, promoting endothelial cell migration, tubulogenesis, and angiogenesis. MCT1 inhibitors (e.g., CHC) block lactate uptake, inhibiting HIF-1 activation and angiogenesis. *In vivo*, MCT1 inhibition reduces tumor vascular density, retarding tumor growth by disrupting lactate-induced pro-angiogenic signaling in the tumor microenvironment [52]. The hypoxic and acidic microenvironment created by lactate accumulation enhances endothelial cell migration and new vessel formation, ensuring nutrient supply and waste removal for the rapidly growing tumor [53], 54].

Moreover, lactate facilitates cancer cell invasion and metastasis by promoting EMT, a process wherein epithelial cells acquire mesenchymal properties, increasing motility and invasiveness. Mechanistically, lactate activates signaling pathways such as PI3K/AKT, NF-κB, and TGF-β, all of which are involved in the induction of EMT and metastatic progression [55], [56], [57]. Lactate-induced EMT also enhances the resistance of cancer cells to apoptosis and facilitates their escape from the primary tumor site.

Collectively, these findings underscore the multifunctional role of lactate in tumor biology. By simultaneously modulating metabolic, immunologic, and structural aspects of the TME, lactate acts as a central driver of tumor progression and a promising therapeutic target in GI cancers.

## 3 Regulatory role of circRNAs in lactate metabolism

CircRNAs have emerged as pivotal regulators of cancer metabolic reprogramming, particularly in glycolysis and lactate metabolism, which are essential for sustaining tumor growth. Their covalently closed-loop structure confers remarkable stability and resistance to exonuclease degradation, enabling long-lasting regulatory effects [58]. CircRNAs exert their influence through multiple mechanisms, including miRNA sponging, interaction with RNA-binding proteins, modulation of transcription, and in certain cases translation into functional peptides. These versatile actions allow circRNAs to control the expression of glycolytic enzymes, lactate transporters, and lactylation-related factors, thereby linking metabolic adaptation to oncogenic progression.

### 3.1 Coordinated regulation of the glycolysis-lactate axis by circRNAs

CircRNAs modulate lactate metabolism primarily through miRNA sponging, whereby they sequester miRNAs that normally repress metabolic genes. In this manner, circRNAs relieve miRNA-mediated inhibition of glycolytic enzymes, such as LDHA. For example, circPDCD11 and circCSNK1G1 enhance LDHA expression and thereby promote the conversion of pyruvate to lactate [59], 60]. Similarly, circ_0069094 and circ_0001955 upregulate HK2 and GLUT1, thereby accelerating glycolytic flux [61], 62]. These findings indicate that circRNAs function as upstream regulators of the enzymatic machinery driving lactate production.

In addition to regulating glycolytic enzymes, circRNAs influence lactate export by modulating transporter expression and stability. For instance, circSIPA1L3 interacts with RNA-binding proteins to stabilize MCT1 mRNA, facilitating efficient lactate export and preventing intracellular acidification [63]. Such regulation enables tumor cells to maintain metabolic homeostasis while sustaining high glycolytic activity.

Some circRNAs further support metabolic adaptation under hypoxic conditions by activating transcription factors such as HIF-1α, either directly or through upstream signaling pathways. This activation increases LDHA transcription and supports continuous lactate production even in oxygen-deprived environments [64], 65]. Collectively, these multi-layered regulatory mechanisms-spanning enzyme expression, transporter function, and hypoxia adaptation-illustrate how circRNAs integrate transcriptional and post-transcriptional control to fine-tune the glycolysis-lactate axis, thereby shaping a tumor metabolic phenotype optimized for survival and progression.

### 3.2 Oncogenic consequences of circRNA-driven lactate reprogramming

CircRNAs that promote lactate production and export exert profound effects on the TME, reshaping its metabolic and cellular landscape to favor tumor progression. Elevated lactate acidifies the extracellular space, which suppresses cytotoxic T cell activity and drives macrophage polarization toward the pro-tumorigenic M2 phenotype [66], 67]. This immunosuppressive state not only weakens anti-tumor immunity but also creates conditions conducive to tumor cell survival and expansion. Thus, circRNA-mediated lactate accumulation serves as a key mechanism of immune evasion within the TME.

Beyond immune modulation, lactate acts as a substrate for histone lactylation, an epigenetic modification that can activate oncogenes, enhance cancer stemness, and promote EMT [68]. CircRNAs that increase LDHA or MCT1 expression indirectly enhance lactylation activity, thereby reinforcing transcriptional programs linked to metastasis and therapy resistance. Through this mechanism, circRNAs couple metabolic output with chromatin remodeling to establish durable transcriptional reprogramming in tumor cells.

CircRNAs also interact with major oncogenic signaling pathways, including PI3K/AKT, HIF-1α, and NF-κB, thereby sustaining pro-survival and pro-metastatic states. For instance, circDENND4C and circRPN2 upregulate glycolytic enzymes and glucose transporters, boosting lactate output while simultaneously amplifying signaling cascades that promote invasion and drug resistance [69]. Through this convergence of metabolic, epigenetic, and signaling networks, circRNAs act as central integrators of malignant potential, highlighting their value as therapeutic targets for disrupting tumor aggressiveness.

Together, circRNAs regulate glycolysis and lactate metabolism through conserved mechanisms, including miRNA sequestration and modulation of metabolic enzymes. However, the architecture and dominant nodes of these regulatory networks differ across gastrointestinal cancers, reflecting tumor-specific metabolic dependencies and microenvironmental constraints. Understanding these context-dependent patterns is essential for delineating the distinct roles of circRNAs in individual GI cancers.

## 4 Differential roles of CircRNAs in lactate metabolism across GI cancers

Although circRNAs regulate lactate metabolism through conserved mechanisms, the structure and regulatory hierarchy of these networks differ across GI cancers. Tumor-specific metabolic requirements, hypoxic gradients, and dominant oncogenic pathways determine how circRNA circuits couple glycolytic enzyme control with downstream signaling programs. Comparative analysis across hepatocellular, gastric, colorectal, esophageal, and pancreatic cancers therefore reveals distinct regulatory architectures underlying lactate-driven tumor progression (Table 1).

**Table 1:** Overview of circRNAs regulation lactate metabolism across GI cancers.

circRNA	Regulation	Target miRNA	Target gene(s)	Cancer type	Functional impact
circROBO1	Up	miR-130a-5p	ROBO1, CCNT2	HCC	Enhances glycolysis and lactate production
circ_0078710	Up	miR-431-5p	TXNDC5	HCC	Promotes glucose uptake and lactate output
circKIF4A	Up	miR-335	ALDOA, HK2, PKM2	HCC	Drives metabolic reprogramming
circFBLIM1	Up	miR-338	LRP6	HCC	Increases ECAR and lactate secretion
circ_0031242	Up	miR-944	MAD2L1	HCC	Promotes glycolysis and proliferation
circZFR	Up	miR-375	HMGA2	HCC	Elevates ECAR and ATP levels
hsa_circ_0001806	Up	miR-125b	HK2	HCC	Enhances glycolytic flux
circ_0091579	Up	miR-490-5p	CASC3	HCC	Promotes glucose consumption
circ_MAPK9	Up	miR-642b-3p	STAT3, LDHA	HCC	Reinforces aerobic glycolysis
circ-PRKCI	Up	miR-1294/miR-186-5p	FOXK1, HK2	HCC	Strengthens glycolytic enzyme expression
circ-PRMT5	Up	miR-188-5p	HK2	HCC	Enhances glycolysis
mcPGK1	Up	–	PGK1	HCC	Shifts OXPHOS to glycolysis
circMAT2B	Up	miR-338-3p	PKM2	HCC	Promotes hypoxia-driven glycolysis
circPVT1	Up	miR-377	TRIM23	HCC	Enhances glycolysis and tumor growth
circ_0004913	Down	miR-184	HAMP	HCC	Suppresses glycolysis
circDNMT1	Up	miR-576-3p	HIF-1α	GC	Promotes hypoxia-adaptive glycolysis
circ-DONSON	Up	miR-149-5p	LDHA	GC	Increases lactate production
circ_0009910	Up	miR-361-3p	SNRPA	GC	Enhances ECAR
circ_0032821	Up	miR-1236-3p	HMGB1	GC	Boosts glycolytic flux
circBFAR	Up	miR-513a-3p	HK2	GC	Reinforces glycolysis
circZNF131	Up	miR-186-5p	HK2	GC	Promotes glucose phosphorylation
circFLNA	Up	miR-1200/miR-646	PFKFB2	GC	Increases glycolytic rate
circ_0000592	Up	miR-1179	ANXA4	GC	Enhances lactate output
circSLAMF6	Up	miR-204-5p	MYH9	GC	Supports hypoxia adaptation
circC6orf132	Up	miR-873-5p	PRKAA1	GC	Promotes metabolic adaptation
circ-MAT2B	Up	miR-515-5p	HIF-1α	GC	Sustains glycolytic signaling
circ_0000419	Down	miR-300	RGMB	GC	Reduces glucose uptake
circTATDN3	Up	miR-511-5p	LDHA	CRC	Boosts lactate production
circHIF1A	Up	miR-361-5p	HIF-1α, LDHA	CRC	Promotes glycolysis and resistance
circTUBGCP3	Up	miR-375	ROCK1	CRC	Increases glycolytic flux
circ-IGF1R	Up	miR-362-5p	HMGB3	CRC	Activates Wnt-driven metabolism
hsa_circ_0045932	Up	miR-873-5p	HK2	CRC	Enhances glycolysis
circ_0087862	Up	miR-296-3p/miR-512-3p	PGK1, HK2	CRC	Multi-node glycolytic activation
circDENND4C	Up	miR-760	GLUT1	CRC	Increases glucose uptake
circ_0053277	Up	miR-520h	HK1	CRC	Promotes glycolysis
circAGFG1	Up	miR-7-5p	PKM2	CRC	Enhances Warburg effect
circTADA2A	Down	miR-374a-3p	KLF14	CRC	Suppresses glycolysis
circ_0094343	Down	miR-766-5p	TRIM67	CRC	Reduces lactate production
circCDC6	Down	miR-3187-3p	PRKAA2	CRC	Inhibits anabolic metabolism
circFAM120B	Down	miR-645	TGFBR2	CRC	Suppresses glycolytic signaling
circ_0003340	Up	miR-874-3p	ENAH	EC	Promotes glycolysis
circRNA6448-14	Up	miR-455-3p	OTUB2	EC	Enhances glucose uptake
circGOT1	Up	miR-606	GOT1	EC	Facilitates aerobic glycolysis
circ_0001944	Up	miR-338-5p	PDK1	EC	Increases lactate production
circ_0006948	Up	miR-3612	HK2, LDHA	EC	Reinforces glycolytic enzymes
hsa_circ_0006168	Up	miR-384	RBBP7	EC	Activates metabolic signaling
circLPAR3	Up	miR-873-5p	LDHA	EC	Promotes Warburg effect
circFNDC3B	Up	miR-490-5p	TXNRD1	EC	Enhances glycolysis
circ_0086414	Down	miR-1290	SPARCL1	EC	Suppresses glucose consumption
circ_0099999	Up	miR-330-5p	FSCN1	PC	Enhances glycolysis
circSLIT2	Up	miR-510-5p	c-Myc, LDHA	PC	Activates LDHA transcription
circ_0072088	Up	miR-545-3p	SLC7A11	PC	Increases glucose uptake
circR3HCC1L	Up	miR-873-5p	PKM2	PC	Promotes hypoxia-driven glycolysis
hsa_circ_0012634	Down	miR-147b	HIPK2	PC	Suppresses glycolysis

### 4.1 Hepatocellular carcinoma: hypoxia-STAT3-stemness integration

HCC exhibits pronounced metabolic plasticity, enabling tumor cells to adapt to hypoxia, nutrient limitation, and therapeutic stress. In this setting, circRNAs act as metabolic amplifiers that integrate glycolytic activation with oncogenic signaling and stemness maintenance. Rather than merely elevating lactate output, circRNA-driven metabolic rewiring in HCC converges on hypoxia-responsive transcription factors, STAT3 signaling, and tumor-initiating cell programs, thereby coupling bioenergetic reprogramming to aggressive phenotypes.

Multiple oncogenic circRNAs enhance glycolysis through ceRNA-mediated derepression of metabolic regulators. circROBO1 promotes glucose uptake and lactate accumulation via the miR-130a-5p/ROBO1 [70] and CCNT2 [71] axes, reinforcing the Warburg phenotype and tumor growth. circFBLIM1 [72], abundant in HCC serum exosomes, sponges miR-338 to upregulate LRP6, increasing glucose consumption, lactate output, ATP levels, and extracellular acidification rate (ECAR). circ_0031242 [73] and circZFR [74] activate the miR-944/MAD2L1 and miR-375/HMGA2 axes, respectively, enhancing glucose consumption, lactate production and promoting proliferation and invasion. hsa_circ_0001806 [75] upregulates HK2 via miR-125b sponging, circ_0091579 [76] elevates CASC3 through miR-490-5p inhibition-all driving aerobic glycolysis and HCC progression. circ_0078710 [77] activates the miR-431-5p/TXNDC5 pathway to increase glycolytic flux and ATP production, whereas circKIF4A [78] drives metabolic reprogramming through the miR-335/ALDOA/OCT4 axis, elevating HK2 and PKM2 expression to sustain proliferation and metastatic adaptation.

Under hypoxic conditions, circRNAs further consolidate glycolytic dominance. circMAT2B sponges miR-338-3p to upregulate PKM2, thereby enhancing glucose uptake and lactate production and promoting HCC progression through a hypoxia-responsive circMAT2B/miR-338-3p/PKM2 axis [79]. Complementing this mechanism, circPVT1 augments glycolysis and tumor growth by relieving miR-377-mediated repression of TRIM23 [80], thereby promoting proliferation while suppressing apoptosis and reinforcing lactate-producing metabolism. In parallel, circ_MAPK9 [81] enhances aerobic glycolysis via miR-642b-3p-dependent activation of signal transducer and activator of transcription 3 (STAT3) and LDHA, linking metabolic reprogramming to inflammatory and survival signaling.

CircRNAs also connect glycolysis to stemness circuitry. circ-PRKCI [82] and circ-PRMT5 [83] upregulate forkhead box K1(FOXK1) and HK2, respectively, increasing expression of HK2, GLUT1, and LDHA to strengthen glycolytic throughput. Notably, the mitochondria-encoded circRNA mcPGK1 [84] facilitates mitochondrial import of PGK1 via TOM40, activating the PGK1-PDK1-PDH axis to shift metabolism toward glycolysis. The resulting lactate accumulation activates Wnt/β-catenin signaling and supports liver tumor-initiating cell self-renewal, directly linking metabolic remodeling to stemness maintenance.

Conversely, circ_0004913 [85] is downregulated in HCC and sponges miR-184 to upregulate HAMP, reducing glucose consumption, lactate production, and ATP levels while suppressing JAK2/STAT3/AKT signaling. This metabolic attenuation impairs proliferation and invasion, ultimately inhibiting tumor growth *in vivo*.

Collectively, circRNA-mediated metabolic regulation in HCC integrates hypoxia adaptation, inflammatory signaling, apoptotic control, and stemness maintenance. By sustaining lactate dominance while reinforcing oncogenic transcriptional networks, circRNAs establish a self-reinforcing metabolic-signaling axis that underpins tumor aggressiveness and therapeutic resistance.

### 4.2 Gastric cancer: hypoxia persistence-chemoresistance-exosomal niche reinforcement

In GC, circRNA-mediated lactate regulation is prominently linked to hypoxia persistence, therapeutic resistance, and tumor-microenvironment communication. Rather than acting as isolated enzyme modulators, GC-associated circRNAs are frequently embedded within feedback-driven metabolic networks that stabilize lactate production under fluctuating oxygen and treatment conditions.

Several circRNAs in GC function as hypoxia-responsive metabolic stabilizers that consolidate lactate-producing phenotypes under oxygen-limited conditions. circDNMT1 [86] enhances HIF-1α-associated transcriptional activity, positioning it within the hypoxia arm of the circRNA-lactate axis and linking metabolic activation to invasive potential. circ-DONSON [87], by elevating LDHA expression, strengthens lactate accumulation while simultaneously promoting angiogenesis and reducing radiosensitivity, illustrating how metabolic amplification in GC frequently coexists with therapy resistance.

Other circRNAs-including circ_0009910 [88] and circ_0032821 [89]-operate within regulatory networks that coordinate glycolytic enhancement with RNA-processing and chromatin-associated factors (SNRPA, HMGB1), suggesting that metabolic reprogramming in GC is embedded within broader transcriptional control systems rather than isolated enzymatic upregulation. circBFAR [90], circZNF131 [91], circFLNA [92], 93],and circ_0000592 [94] further reinforce high-flux glycolysis by synchronizing upstream rate-limiting steps (HK2, PFKFB2, Annexin A4) with proliferative signaling cascades, thereby consolidating sustained lactate output as a structural feature of GC metabolism.

Hypoxia-adaptive circRNAs represent a particularly prominent subset in GC. circSLAMF6 [95] and circC6orf132 [96] enhance metabolic resilience under low oxygen tension, while circ-MAT2B [97] forms a positive regulatory loop with HIF-1α, stabilizing glycolytic commitment and maintaining persistent lactate secretion. This feedback-driven architecture highlights how GC circRNAs not only activate glycolysis but also preserve its dominance in fluctuating microenvironmental conditions.

Beyond intrinsic tumor metabolism, GC-specific circRNAs frequently extend their influence into the tumor ecosystem. circ_0006089 [98] integrates glycolytic activation with TGFB1-mediated metastatic signaling, and circLDLR [99] links metabolic rewiring to chromatin remodeling via CHD1. circVPS33B [100] and circ-NRIP1 [101], 102] further demonstrate how lactate regulation in GC converges with EMT and chemoresistance pathways, particularly through HIF-1α-dependent 5-fluorouracil resistance mechanisms. Notably, exosomal circKIAA1797 [103] exemplifies a GC-specific feature: metabolic information transfer to surrounding cells, facilitating the establishment of a lactate-enriched microenvironment conducive to tumor expansion.

In contrast, downregulated circRNAs in GC illustrate the vulnerability of this metabolic circuitry. circ_0000419 [104] and circ_0004872 [105] attenuate glycolytic dominance by limiting glucose utilization and lactate secretion, thereby weakening invasive capacity. These tumor-suppressive circRNAs underscore that lactate-driven metabolic programming in GC is not merely an enzymatic phenomenon but a networked state that can be destabilized through circRNA modulation.

Collectively, GC is distinguished by circRNA networks that tightly couple lactate metabolism to hypoxia persistence, epigenetic adaptation, and therapy resistance. Rather than functioning solely as metabolic enhancers, GC-associated circRNAs act as regulatory hubs that stabilize a lactate-dependent malignant phenotype under therapeutic and microenvironmental stress.

### 4.3 Colorectal cancer: Wnt coupling-kinase resistance-multi-node glycolytic reinforcement

CRC is characterized by remarkable metabolic adaptability, in which circRNA-regulated lactate production is closely intertwined with Wnt/β-catenin signaling, kinase-driven proliferation, and EGFR-associated therapeutic resistance. In this context, circRNAs function as integrative nodes that synchronize metabolic flux with oncogenic signaling cascades.

A distinctive characteristic of CRC is the integration of lactate metabolism with canonical Wnt signaling. circ-IGF1R [106] activates the miR-362-5p/HMGB3 axis to enhance Wnt/β-catenin activity, situating glycolytic reinforcement within a stemness-supportive transcriptional environment. Similarly, circTUBGCP3 [107] links metabolic activation to ROCK1-mediated cytoskeletal remodeling, underscoring how circRNA-driven lactate production in CRC often coexists with enhanced migratory and invasive potential.

circTATDN3 [108] exemplifies a dual-layer metabolic control strategy in CRC. Beyond miRNA-mediated regulation of LDHA expression, it directly interacts with LDHA to enhance its phosphorylation and enzymatic activity, demonstrating that CRC circRNAs may operate simultaneously at transcriptional and post-translational levels. This layered regulation highlights a CRC-specific pattern in which metabolic amplification is reinforced through structural enzyme modulation rather than simple gene depression.

Resistance-associated metabolic rewiring is another prominent CRC feature. circHIF1A [109] strengthens hypoxia-related transcriptional networks that elevate GLUT1 and LDHA expression, contributing to cetuximab resistance. circDENND4C [110], circ_0053277 [111], and circAGFG1 [112] similarly coordinate glycolytic reinforcement with pro-survival signaling cascades, linking lactate metabolism to therapy evasion rather than merely proliferation.

Some CRC circRNAs function as multi-node regulators within the glycolytic network. circ_0087862 [113], 114] simultaneously modulates PGK1 and HK2 through distinct miRNA axes, illustrating how CRC metabolic circuitry often relies on parallel reinforcement of upstream and downstream glycolytic checkpoints. hsa_circ_0045932 [115] further integrates HK2 activation into broader proliferative programs, consolidating glycolytic commitment as a stable metabolic state.

Conversely, tumor-suppressive circRNAs in CRC reveal that this metabolic configuration remains reversible. circTADA2A [116], circ_0094343 [108], circCDC6 [117], and circFAM120B [118] attenuate glycolytic dominance through AMPK activation, TRIM67 modulation, and TGFBR2-associated pathways. Notably, exosomal circ_0094343 [119] restores chemosensitivity, illustrating that lactate metabolism in CRC is tightly linked to drug response dynamics and can be therapeutically destabilized.

Overall, CRC is characterized by circRNA networks that tightly interlock lactate metabolism with Wnt-driven stemness, kinase-mediated resistance pathways, and multi-layer enzymatic reinforcement. Rather than acting solely as metabolic enhancers, CRC-associated circRNAs function as integrative hubs that synchronize glycolytic flux with proliferative signaling and therapeutic adaptation.

### 4.4 Esophageal cancer: metabolic intensification-therapeutic evasion-intercellular amplification

Esophageal cancer (EC) exhibits a metabolically aggressive phenotype characterized by sustained aerobic glycolysis and high lactate turnover. In this malignancy, circRNA networks function primarily as metabolic intensifiers, reinforcing glycolytic flux and integrating lactate production with invasive growth and therapeutic resistance.

Several upregulated circRNAs in EC consolidate this high-flux metabolic state. circ_0003340 [120] enhances ENAH-associated proliferative signaling while strengthening glucose consumption and lactate output, positioning it within a coordinated invasion-metabolism axis. circRNA6448-14 [121] amplifies glycolytic commitment through the miR-455-3p/OTUB2 pathway, linking lactate reinforcement to cancer stemness features in esophageal squamous cell carcinoma (ESCC). Similarly, circGOT1 [122] connects host gene activation with metabolic adaptation and cisplatin resistance, illustrating how lactate regulation in EC frequently aligns with therapeutic evasion mechanisms.

A defining feature of EC-associated circRNAs is their coordination of glycolytic enzyme activation with broader oncogenic signaling pathways. circ_0001944 [123] facilitates PDK1-driven metabolic shifting toward glycolysis, thereby strengthening invasive and migratory phenotypes. circ_0006948 [124] and circLPAR3 [125] reinforce LDHA-associated lactate production, consolidating the Warburg phenotype as a structural metabolic backbone of EC progression. hsa_circ_0006168 [126] further integrates metabolic amplification with S6K/S6 pathway activation, embedding lactate regulation within translational control and growth signaling networks.

The microenvironmental dimension of EC metabolism is also shaped by circRNA-mediated intercellular communication. Exosomal circFNDC3B [127] extends glycolytic influence beyond tumor-intrinsic circuitry, promoting proliferation and tumor growth *in vivo*. In contrast, circ_0086414 [128] attenuates this amplification state by reducing lactate output and suppressing proliferative capacity, underscoring the regulatory reversibility of circRNA-driven metabolic programs.

Collectively, EC is distinguished by circRNA circuits that intensify glycolytic throughput and synchronize lactate production with invasion, stemness, and chemoresistance. Rather than operating as isolated enzyme regulators, EC-associated circRNAs function as metabolic accelerators embedded within aggressive oncogenic signaling environments.

### 4.5 Pancreatic cancers: metabolic rigidity-persistent lactate dominance-hypoxia reinforcement

Pancreatic cancer represents an extreme example of glycolytic dependency, where circRNA networks reinforce a persistently lactate-driven metabolic state. Unlike more dynamically regulated tumors, PC exhibits a rigid metabolic configuration in which circRNAs stabilize glycolytic dominance across diverse microenvironmental conditions.

Circ_0099999 [129] exemplifies this rigid metabolic configuration by enhancing FSCN1-associated proliferative signaling while consolidating glucose consumption and lactate secretion. circSLIT2 [130] integrates circRNA regulation with the c-Myc transcriptional program, reinforcing LDHA expression and sustaining glycolytic commitment. Similarly, circ_0072088 [131] coordinates increased extracellular acidification rate (ECAR) with reducing oxygen consumption rate, illustrating how PC circRNAs stabilize glycolytic dominance rather than transiently activating it.

Hypoxia further intensifies this regulatory architecture. Hypoxia-induced exosomal circR3HCC1L [132] strengthens PKM2-associated metabolic flux, promoting tumor growth across oxygen gradients and reinforcing metabolic adaptability within the PC microenvironment. Conversely, has_circ_0012634 [133] demonstrates that suppression of this rigid glycolytic state can destabilize PC cell proliferation. By activating HIPK2 and attenuating lactate production, it weakens the metabolic foundation supporting tumor progression.

Collectively, PC circRNA networks are characterized by reinforcement of metabolic rigidity and sustained lactate output, distinguishing pancreatic cancer from other GI malignancies where circRNA-lactate regulation may be more dynamically modulated.

## 5 Potential clinical applications and therapeutic implications

The discovery of circRNAs as regulators of lactate-centered metabolic reprogramming in GI cancers opens new avenues for clinical applications in diagnostics, prognostics, and targeted therapies. However, clinical deployment will require a clearer definition of the relevant regulatory networks, together with solutions to key technological and biological constraints.

### 5.1 CircRNAs as biomarkers

CircRNAs are covalently closed, exonuclease-resistant RNA molecules with high stability in bodily fluids such as blood, saliva, and urine. Their cancer-specific expression profiles and spatiotemporal specificity make them attractive candidates as non-invasive biomarkers. Numerous studies have demonstrated that plasma-derived circRNAs are differentially expressed in GI cancer patients compared to healthy individuals, suggesting their utility in early diagnosis and disease monitoring [134], 135]. For instance, elevated levels of circHIPK3 and circNRIP1 in gastric cancer, and circZFR in HCC, correlate with tumor burden, lactate dehydrogenase activity, and poor prognosis [74], 136]. Linking circRNA abundance to metabolic phenotypes may further enhance clinical interpretability, as dynamic changes in circRNA levels could reflect shifts in tumor metabolic state.

### 5.2 Therapeutic targeting of CircRNAs

Given their upstream regulatory roles, circRNAs provide unique opportunities for therapeutic modulation. Technologies such as small interfering RNAs (siRNAs), antisense oligonucleotides (ASOs), and CRISPR/Cas13 systems have been developed to knock down oncogenic circRNAs or restore tumor-suppressive ones [79]. Preclinical models have shown that silencing oncogenic circRNAs like circMAT2B and circPVT1 reduces lactate output and restrains tumor growth [137]. However, efficient and tumor-selective delivery, particularly for targeting solid tumors, remains a major limitation. Lipid nanoparticles, viral vectors, and exosome-based delivery platforms are under investigation to improve tumor-specific circRNA targeting [138].

### 5.3 Targeting lactate metabolism in cancer therapy

Several metabolic inhibitors targeting lactate metabolism are currently in preclinical and clinical development. These include LDHA inhibitors (e.g., FX11), MCT1 inhibitors (e. g., AZD3965), and modulators of HIF-1α activity [4], 139]. CircRNA-targeted strategies may synergize with these agents by indirectly modulating the expression of key glycolytic enzymes or transporters. For example, inhibiting circRNAs that regulate LDHA (e.g., circ_0055976, circ_100782) or MCT4 (e.g., circHIPK3) may enhance the efficacy of pharmacological inhibitors by reducing compensatory metabolic fluxes [140]. Moreover, combination therapies involving circRNA silencing and metabolic inhibitors may overcome therapeutic resistance and improve outcomes in metabolically heterogeneous tumors such as CRC and HCC.

### 5.4 Combination therapy strategies

Combination approaches may increase the depth and durability of metabolic intervention by co-targeting circRNA regulatory axes and downstream enzymatic/transport effectors. In particular, circRNA silencing combined with LDHA inhibitors may further reduce lactate production, whereas co-administration with MCT1 inhibitors may limit lactate export and tumor-microenvironment metabolic coupling.

Beyond metabolic control, accumulating evidence indicates that circRNAs also participate in therapeutic resistance programs in gastrointestinal cancers. A recent comprehensive review highlighted the involvement of ncRNAs, including circRNAs, in 5-fluorouracil (5-FU) resistance through modulation of oncogenic pathways such as HIF-1α signaling and metabolic effectors that support tumor survival under chemotherapeutic stress [141]. In colorectal cancer, circNCOA3 has been shown to promote immune escape and resistance to PD-1 blockade via the circNCOA3/miR-203a-3p.1/CXCL1 axis, reshaping the immune microenvironment by reducing CD8^+^ T-cell infiltration and increasing MDSC accumulation [142].

Together, these data support a rationale for integrating circRNA modulation with lactate-pathway inhibitors (LDHA/MCT1), chemotherapy (e.g., 5-FU), or immunotherapy (e.g., PD-1 blockade) to co-suppress metabolic resilience and resistance-associated signaling networks. Such regimens may be particularly relevant in metabolically dependent tumors, including hepatocellular carcinoma and pancreatic ductal adenocarcinoma.

### 5.5 Challenges and future directions

Despite their therapeutic potential, circRNA-based interventions face major challenges, including (i) specificity constraints arising from sequence overlap with linear transcripts and potential off-target effects; (ii) delivery barriers in achieving efficient, tissue-selective exposure in solid tumors; (iii) functional redundancy within regulatory networks that can enable compensatory activation; and (iv) incomplete annotation, as many circRNAs and circRNA-miRNA-mRNA interactomes remain insufficiently mapped. Future work should prioritize high-throughput identification of circRNA-metabolism interactions, development of cancer-type-specific circRNA panels, and validation in patient-derived organoids and xenograft models, while integrating transcriptomic, metabolomic, and epigenomic profiling to define actionable nodes and predict therapeutic response [143].

## 6 Conclusions

In conclusion, circRNAs represent a regulatory layer connecting lactate-centered metabolic reprogramming with key malignant phenotypes in GI cancers, including immune evasion, metastasis, and treatment resistance. Continued mechanistic and translational studies may enable metabolism-informed circRNA targeting that complements existing therapeutic paradigms. Ultimately, circRNA-guided strategies may advance precision oncology by improving biomarker performance and enabling rational combination therapies.
